# Beyond Eosinophil Depletion: IL-5 as a Context-Dependent Regulator of Airway Immune Networks

**DOI:** 10.3390/ijms27094077

**Published:** 2026-05-02

**Authors:** Shih-Lung Cheng

**Affiliations:** 1 Department of Pulmonary Medicine, Far Eastern Memorial Hospital, New Taipei City 22000, Taiwan; femh67139@femh.org.tw; 2 Department of Chemical Engineering and Materials Science, Yuan Ze University, Taoyuan 320315, Taiwan

**Keywords:** eosinophil, IL-5, asthma, COPD

## Abstract

Interleukin-5 (IL-5) has long been positioned as a lineage-restricted cytokine primarily responsible for eosinophil differentiation and survival. However, emerging mechanistic and clinical evidence supports a broader conceptual shift: IL-5 should no longer be viewed solely as an eosinophil growth factor, but as a context-dependent regulator embedded within dynamic airway immune networks. Drawing on advances in eosinophil subset biology, receptor signaling, and tissue-level immune crosstalk, this review reframes IL-5 biology through the lens of systems-level inflammatory regulation across airway and systemic eosinophilic diseases. Recent data reveal functional heterogeneity between resident and inflammatory eosinophil subsets, challenging the assumption that blood eosinophilia uniformly reflects pathogenic activity. In parallel, functional IL-5 receptor expression has been identified on multiple structural and immune cell populations—including epithelial cells, mast cells, plasma cells, basophils, neutrophils, and fibroblasts—supporting a broader tissue-signaling paradigm. Experimental and translational studies further link IL-5 to epithelial integrity, airway remodeling, and mucus pathology, suggesting structural and network-level effects beyond simple eosinophil depletion. Comparative analyses across asthma, chronic rhinosinusitis with nasal polyps, and COPD demonstrate that eosinophilic inflammation is biologically heterogeneous and context-dependent. While IL-5-targeted therapies yield consistent benefit in severe asthma, therapeutic responses in other airway diseases appear to be shaped by local tissue architecture and mixed inflammatory programs. Together, these observations illustrate a paradigm shift from pathway-specific inhibition toward network-informed disease control and highlight key areas for future mechanistic investigation.

## 1. Introduction

Over the past two decades, advances in immunology have progressively reshaped therapeutic development, shifting emphasis from phenotype-driven disease classification toward mechanism-driven intervention [1,2,3]. In this evolving paradigm, key cytokines are increasingly recognized not merely as downstream markers of inflammation, but as upstream coordinators capable of contributing to evolving models of disease stratification and targeted intervention. IL-5 provides a particularly instructive example of this shift [4,5,6].

Although IL-5 has been primarily conceptualized as a regulator of eosinophil differentiation and survival, this framework does not fully capture the breadth of IL-5 biology. Eosinophils themselves display functional heterogeneity across tissues and disease states, and emerging evidence indicates that IL-5 signaling extends to epithelial, stromal, and additional immune cell populations within inflamed airway tissues. Accordingly, this review examines IL-5 across three dimensions: (i) eosinophil biology from homeostasis to activation, (ii) IL-5 signaling beyond eosinophils, and (iii) disease-specific network context across airway and related systemic eosinophilic diseases. Through this lens, IL-5 may function as a context-dependent regulator within tissue immune networks, extending beyond its classical role as a lineage-restricted survival factor.

## 2. From T-Cell-Replacing Factor (TRF) to IL-5: The Discovery of a Pleiotropic Cytokine

IL-5 was initially characterized as a T-cell-derived factor that promoted B-cell differentiation and antibody production, before its role in eosinophil biology became fully appreciated [7,8]. Subsequent molecular and receptor characterization studies unified these activities under a single cytokine, establishing IL-5 as a multifunctional mediator with effects spanning adaptive and innate immune compartments [7,9]. Later work defined its central role in eosinophil differentiation, activation, and survival, which ultimately became the dominant translational focus in airway disease [8,10]. This historical progression underscores that IL-5 biology has long extended beyond eosinophils, even if therapeutic development centered primarily on that axis.

## 3. Eosinophil Biology: From Homeostasis to Pathology

### 3.1. Are Eosinophils Always Harmful?

While early work emphasized eosinophils in allergic inflammation, subsequent studies demonstrated that they are evolutionarily conserved across vertebrate species, implying fundamental biological functions beyond disease states [11]. This conservation suggests that eosinophils participate in physiological processes maintained under steady-state conditions rather than acting solely as pathological effectors. Importantly, many of these baseline functions occur within cytokine environments in which IL-5 contributes to eosinophil development and survival but does not act in isolation.

Under homeostatic conditions, eosinophils are continuously generated in the bone marrow and migrate to peripheral tissues, where their distribution is shaped by coordinated cytokine and chemokine networks, including IL-5 and eotaxins, but is not strictly dependent on IL-5 alone [8,10,12]. Residual eosinophil populations persist in multiple tissues even in IL-5-deficient settings, indicating that tissue residency can be maintained through IL-5-independent mechanisms and local microenvironmental cues [12]. The gastrointestinal tract contains the largest pool of resident eosinophils under non-inflamed conditions, with smaller but stable populations in the lung, adipose tissue, thymus, and reproductive organs. Consistent with this integration, eosinophils support plasma cell survival at mucosal sites and sense microbial signals through pattern-recognition receptors, contributing to immune surveillance without overt inflammation [13,14,15]. These observations underscore that IL-5 operates within a broader regulatory network that sustains eosinophil homeostasis rather than exclusively driving inflammatory amplification.

Beyond immune regulation, accumulating evidence indicates that eosinophils participate in host defense against viral and bacterial pathogens. In respiratory viral infection models, eosinophils recruited to Th2-polarized airways limit viral burden and mortality [16]. Mechanistically, they recognize viral single-stranded RNA through Toll-like receptor 7 (TLR7)–Myeloid Differentiation Primary Response 88 (MyD88) signaling, deploy eosinophil-associated ribonucleases, and engage nitric oxide pathways; adoptive transfer studies demonstrate accelerated viral clearance [17]. Eosinophils also respond to bacterial stimuli by releasing mitochondrial DNA with granule proteins to form extracellular structures that trap and kill bacteria without inducing cell death, conferring protection against sepsis in vivo [18]. Because IL-5 enhances eosinophil survival and functional competence, these host-defense activities further illustrate that IL-5 biology intersects with protective as well as inflammatory programs.

Clinical observations align with these findings. During the 2009 influenza A pandemic, patients with asthma were hospitalized more frequently but experienced lower mortality, paralleling experimental data showing enhanced CD8^+^ T-cell responses and viral clearance in the presence of eosinophils [19]. Profound eosinopenia has also been observed in severe COVID-19, with delayed recovery associated with worse outcomes [20]. Although causality remains unresolved, these patterns suggest that eosinophils—and by extension the cytokine networks that sustain them—may contribute to antiviral immunity rather than merely reflecting inflammatory burden.

### 3.2. What Governs the Shift from “Beneficial” to “Harmful” Eosinophil Behavior Across Tissues and Disease Contexts?

Such context-dependent eosinophil functions are now increasingly understood to reflect the presence of distinct eosinophil populations, commonly described as resident eosinophils (rEOS) under steady-state conditions and inflammatory eosinophils (iEOS) that emerge upon activation [17,21,22,23,24]. Accumulating data now suggest that the transition from rEOS to iEOS is driven less by cytokine exposure alone than by intracellular reprogramming acquired within inflamed tissues [7,25]. Eosinophils isolated from nasal polyps consistently display an activated CD69^hi^ CCR3^low^ CXCR4^−^ Siglec-8^int^ phenotype, accompanied by marked shifts in lipid metabolism, including reduced prostaglandin and 15-lipoxygenase pathways with selective enhancement of leukotriene production, changes that can be reproduced by IL-5, Granulocyte–macrophage colony-stimulating factor (GM-CSF), and innate receptor signaling [26]. Importantly, these metabolic alterations have now been directly linked to eosinophil extracellular trap cell death (EETosis), with long-chain unsaturated fatty acids—particularly arachidonic acid—licensing extracellular trap and Charcot–Leyden crystal release through the IRE1α–XBP1s–PAD4 axis, thereby contributing to steroid-refractory eosinophilic inflammation [27]. In parallel, studies in asthma identify lysosomal remodeling as a distinct activation checkpoint, where increased lysosomal acidity and Cathepsin L activity amplify eosinophil effector functions via arginine metabolism, correlating with disease severity while leaving eosinophil development intact [28,29]. The relative contribution and temporal coordination of these signaling pathways in governing eosinophil state transitions remain incompletely understood and warrant further investigation.

Building on this mechanistic framework, emerging evidence indicates that the balance and functional dominance of rEOS versus iEOS differ substantially between asthma and COPD, with important implications for how eosinophilic inflammation is interpreted across these diseases. In asthma, multiple studies demonstrate preferential enrichment of iEOS programs in both circulation and inflamed tissues, with clear differences in surface phenotype, survival, and IL-5 responsiveness between eosinophil subsets [30,31,32,33] (Table 1). These disease-specific distributions extend the preceding observations by indicating that eosinophil behavior in asthma reflects context-dependent shifts in activation state shaped by local cytokine and tissue cues. Consistent with this view, IL-5 blockade (e.g., mepolizumab) has been reported to be associated with a relative shift from an inflammatory phenotype toward a more homeostatic-like eosinophil profile, suggesting modulation of activation state rather than irreversible lineage reprogramming [32]. Blood-based analyses show that iEOS predominate in steroid-free allergic asthma, whereas severe eosinophilic asthma is characterized by enhanced survival of both rEOS and iEOS, consistent with sustained activation rather than simple numerical expansion [31]. Notably, allergen challenge rapidly reduces circulating iEOS proportions while prolonging their survival, supporting preferential recruitment into inflamed airway compartments rather than depletion [31].

Overview of phenotypic markers, IL-5 dependence, tissue distribution, functional properties, and clinical associations of rEOS and iEOS in human and murine studies. Subset functional assignments are derived from human and murine studies; precise phenotypic boundaries and IL-5 dependence may vary by tissue compartment and disease context. In humans, rEOS are typically CD62L bright with lower IL-5Rα (CD125) expression and exhibit homeostatic/regulatory features, whereas iEOS are CD62L low, express higher IL-5Rα, and display pro-inflammatory activity. In severe eosinophilic asthma, iEOS proportions associate with poorer disease control and exacerbation risk and are reduced by anti-IL-5 therapy, while rEOS predominance correlates with improved clinical response. In COPD, subset distribution and biologic responsiveness remain less clearly defined. Murine lung models similarly distinguish parenchymal, IL-5-independent rEOS from peribronchial, IL-5-dependent iEOS with pro-inflammatory functions, supporting context-dependent eosinophil heterogeneity. As phenotypic definitions and subset distributions vary across species, sampling compartments, and study settings, interpretation should emphasize consistent mechanistic patterns rather than absolute proportions.

Direct comparisons between asthma and COPD further highlight disease-specific eosinophil behavior. Patients with asthma exhibit a markedly higher proportion of circulating iEOS than those with COPD, whereas COPD patients show substantially lower iEOS frequencies despite comparable eosinophilia [30]. In asthma, iEOS express higher levels of IL-5 receptor-α (IL-5Rα) than rEOS, supporting heightened sensitivity to IL-5-driven activation, while in COPD no consistent association is observed between iEOS proportion, corticosteroid use, disease severity, or exacerbation risk [30].

Tissue-based observations extend this distinction. In type 2-associated airway diseases, including asthma-associated CRSwNP, iEOS preferentially accumulate within nasal polyp tissue and often exceed their proportion in peripheral blood, particularly in severe or refractory disease [34]. Circulating iEOS decline following allergen exposure, consistent with rapid tissue migration driven by IL-5, eotaxins, and epithelial alarmins [34]. Functional analyses further suggest that while both rEOS and iEOS contribute to airway smooth muscle interaction and matrix engagement, rEOS display stronger adhesion and migratory capacity, whereas iEOS exhibit greater reactive oxygen species production and inflammatory effector activity [31,34].

However, eosinophils do not fully explain IL-5 biology. Clinical interpretations that center on eosinophil depletion incompletely capture its broader functions, as profound reductions in blood and tissue eosinophils have not consistently translated into clinical improvement across multiple eosinophilic airway diseases [35,36,37,38]. This dissociation supports viewing IL-5 as a coordinator within a broader epithelial–immune network and examining where different IL-5-targeted strategies may diverge mechanistically [39]. If IL-5′s clinical footprint reflects network-level biology, the airway epithelium—shaping barrier integrity and downstream inflammatory circuits—represents a logical point of focus [40].

## 4. IL-5 Beyond Eosinophils: Expanding the Airway Network

What evidence supports roles for IL-5 within a broader airway cellular network? In severe asthma, disease mechanisms arise from the convergence of epithelial barrier dysfunction, immune imbalance, mucus plugging, and airway remodeling—processes that collectively create multiple biological contexts in which IL-5 may exert influence beyond regulating eosinophil number or persistence [40]. Emerging in vitro and in vivo evidence indicates that IL-5 exerts direct effects across multiple structural and immune cell types, extending well beyond its long-recognized role in eosinophil differentiation and survival.

Evidence from both experimental systems and patient-derived tissues increasingly points to a direct role for IL-5 in shaping airway epithelial behavior, independent of its canonical effects on eosinophils [41,42]. Studies using differentiated human bronchial epithelial cells have shown that the epithelium expresses a functional IL-5 receptor complex, with IL-5Rα prominently localized to the apical surface of ciliated cells rather than mucus-producing goblet cells, defining a discrete epithelial compartment that is directly responsive to IL-5 signaling [41,42]. Engagement of this receptor activates intracellular signaling pathways, including Extracellular signal-Regulated Kinase (ERK) and Protein Kinase B/PKB (Akt), and leads to sustained transcriptional changes after prolonged exposure, indicating that epithelial IL-5 signaling is biologically active rather than incidental [41]. Consistent with these molecular effects, in vitro studies using human ciliated airway epithelial cells show that IL-5 directly increases ciliary beat frequency, supporting a functional epithelial response that may enhance mucociliary clearance through mechanisms distinct from eosinophil-driven inflammation [43].

Functionally, IL-5 exposure alters epithelial programs that are central to barrier integrity and tissue resilience. Key genes involved in cell–cell adhesion, tight junction maintenance, and cytoskeletal organization—such as CDH1(E-cadherin), Epidermal Growth Factor Receptor (EGFR), integrins, and caveolins—are consistently downregulated, while immune and stress-response pathways are reshaped in parallel [41]. These changes closely resemble epithelial abnormalities described in asthma and CRSwNP, where impaired barrier function and defective repair contribute to chronic inflammation and remodeling [40]. Importantly, these effects have been demonstrated in epithelial systems without eosinophils, underscoring that IL-5 can act directly on the airway lining rather than exclusively through eosinophil-mediated injury [41,44]. While indirect epithelial effects arising from eosinophil-driven amplification of chronic inflammation likely coexist in vivo [45,46], the available evidence indicates that IL-5 signaling within epithelial cells constitutes a biologically autonomous and functionally relevant pathway.

The epithelium is not only a target of IL-5 but also a local source. Both primary and immortalized human airway epithelial cells constitutively express IL-5 mRNA, with expression further enhanced by inflammatory cues and epithelial injury [44]. Crucially, the functional relevance of epithelial-derived IL-5 has been directly interrogated in murine models using IL-5-deficient bone marrow chimeras that restrict IL-5 expression to stromal compartments, including bronchial epithelial cells [47]. In these systems, epithelial-restricted IL-5 production was sufficient to drive key features of allergic airway disease—such as mucous metaplasia, airway eosinophilia, and antigen-specific IgA responses—even in the absence of hematopoietic IL-5, thereby minimizing confounding contributions from eosinophil-derived cytokine pools and demonstrating a compartmentalized, epithelium-intrinsic IL-5 axis within the airway microenvironment [47]. The evidence indicates that epithelial IL-5 is not merely a by-product of inflammation but can actively shape local immune and barrier responses in the airway independent of systemic IL-5 sources [47].

Clinical and translational observations reinforce the relevance of this epithelial pathway. Recent multi-omic studies show that anti-IL-5 therapy (mepolizumab) induces reproducible, biologically meaningful changes within the airway epithelium that extend beyond suppression of eosinophilic inflammation [48]. Analysis of nasal epithelial methylome and transcriptome profiles in asthma identified a responder signature marked by downregulation of pathways linked to epithelial apoptosis, oxidative stress, and peripheral neutrophil activation, alongside coordinated upregulation of intracellular assembly programs governing microtubule dynamics, cytoplasmic organization, and cytoskeletal structure—core processes underlying epithelial integrity and repair [48].

Nevertheless, network-level analyses indicate that epithelial responses to IL-5 neutralization are complex and context-dependent. In the MUPPITS-2 (Mechanisms Underlying Asthma Exacerbations Prevented and Persistent with Immune-Based Therapy: a Systems Approach Phase 2) cohort, upper-airway transcriptomic profiling revealed that while eosinophil-associated inflammatory modules declined during mepolizumab therapy, multiple epithelial-linked and non-type 2 inflammatory gene modules increased in expression [49]. Modular correlation analyses further demonstrated selective decoupling of eosinophil-specific gene networks, with relative preservation—or strengthening—of co-expression among mast cell-associated genes, type 2 cytokines, and epithelial inflammatory programs [49]. Together, these findings suggest that airway epithelial–immune crosstalk involves additional layers of complexity not yet fully delineated, underscoring the need for deeper mechanistic insight to refine biologic treatment strategies.

### 4.1. Do Immune Cells Beyond Eosinophils Express IL-5 Receptors and Directly Respond to IL-5 in a Manner That Shapes Airway Inflammation?

By reshaping epithelial integrity and stress responses, IL-5 is well placed to modulate the signals that the airway epithelium delivers to underlying immune cells, including alarmins, cytokines, and chemokines that govern immune recruitment and polarization [40,41,50]. This epithelial–immune interface provides a logical bridge between IL-5 signaling at the tissue surface and broader alterations in airway immune balance, setting the stage for understanding how IL-5 shapes inflammatory networks beyond eosinophils [51,52]. While ILC2s are recognized as principal IL-5-producing cells in type 2 airway inflammation [53,54], the emphasis here is on downstream IL-5 responsiveness across additional airway cell populations.

Across multiple studies in severe eosinophilic asthma, both IL-5 ligand neutralization and IL-5Rα-directed therapies converge on a common immunologic outcome: rebalancing of adaptive immune compartments. Treatment with either mepolizumab or benralizumab is consistently associated with a reduction in effector memory CD4^+^ T cells alongside an expansion of regulatory T cells, changes that correlate with improved lung function, asthma control, and reduced corticosteroid dependence [55,56]. These parallel effects, observed despite fundamentally different mechanisms of action, suggest that modulation of immune balance might be a shared downstream consequence of interrupting IL-5-dependent immune networks within a broader type 2 inflammatory milieu rather than a simple reflection of eosinophil depletion.

Where these strategies begin to diverge is in their ability to engage the broader network of IL-5-responsive immune cells present within inflamed airway tissues. Beyond eosinophils, multiple immune populations express functional IL-5 receptors and respond directly to IL-5 signaling, including mast cells, plasma cells, basophils, and subsets of innate and adaptive immune cells [9,39,42,57,58]. Recent mechanistic studies show that IL-5 directly enhances mast cell antiviral function, increasing type I and III interferon production while promoting cell survival through upregulation of pro-survival pathways such as B-cell lymphoma 2 (BCL2) and endothelial PAS domain protein 1 (EPAS1) [59]. Blockade of IL-5 is associated with reduced EPAS1 expression in patients receiving anti-IL-5 therapies, indicating that IL-5 signaling helps maintain mast cell viability and function under inflammatory stress [59]. These findings position mast cells as IL-5-responsive effector cells regulated independently of eosinophils.

Mast cells, another example, are not only targets of IL-5 signaling but can also act as a local source of IL-5 following IgE receptor engagement, with rapid induction of IL-5 transcripts and protein after activation [60]. Human mast cells express functional IL-5Rα, and IL-5 stimulation enhances their survival, proliferation, and activation state, particularly under inflammatory or viral stress [39]. In upper-airway disease, mast cells within nasal polyp tissue express IL5RA transcripts and contribute to local production of lipid mediators such as Prostaglandin D2 (PGD_2_) and cysteinyl leukotrienes, processes amplified by IL-5 and attenuated by IL-5 neutralization, even when tissue eosinophil numbers are not substantially reduced [58].

IL-5 also acts directly on local antibody-producing cells within nasal polyp tissue, particularly in aspirin-exacerbated respiratory disease (AERD) [57,61]. Plasma cells and antibody-secreting cells isolated from CRSwNP tissue exhibit upregulated and functionally active IL-5Rα, and stimulation with IL-5 induces coordinated transcriptional changes, including activation of proliferative programs such as cyclin D2, as well as immunoglobulin-related transcripts linked to IgE production [57,61]. This IL-5-responsive antibody program appears to be largely tissue-restricted and differs from classical germinal center-driven IgE generation, which relies primarily on IL-4 and IL-13 signaling and is reflected in systemic IgE levels [57]. Within the nasal mucosa, extrafollicular IgD^+^ B cells progressively acquire an IL-5-dominant signaling profile as they differentiate into antibody-secreting cells, characterized by loss of IL-4 receptor expression and increasing IL-5Rα expression [57,61]. In this context, IL-5 functions as a local survival and activation signal for IgE-producing cells, consistent with observations that tissue IgE levels correlate with nasal polyp regrowth and disease recurrence in AERD [61].

Direct IL-5 responsiveness extends to other granulocyte populations within the airway. Human basophils express functional IL-5 receptors, and IL-5 stimulation enhances histamine release and activation independently of eosinophils [9]. Airway neutrophils likewise express IL-5Rα at levels exceeding those in peripheral blood, underscoring the importance of the local airway microenvironment in shaping IL-5 sensitivity [39]. Ex vivo IL-5 stimulation induces phenotypic changes consistent with neutrophil activation, and airway neutrophil IL-5Rα expression correlates more closely with viral presence than with eosinophil burden in severe asthma, further dissociating IL-5 biology from circulating eosinophil counts alone [39]. These findings support a model in which IL-5 signaling operates within a tissue-restricted immune network involving mast cells, basophils, neutrophils, and innate immune pathways, such that modulation of IL-5 activity in diseased airways may not be adequately reflected by blood-based biomarkers.

By contrast, IL-5Rα-directed antibody-dependent cellular cytotoxicity (ADCC) strategies rely on adequate surface receptor density and intact Fc-mediated cytotoxic effector mechanisms to achieve cell depletion [62,63]. Benralizumab effectively depletes tissue eosinophils and basophils via ADCC [62,64]. Despite this depletion, studies in atopic dermatitis show limited clinical benefit, potentially because the drug spares mast-cell populations and leaves significant T-cell compartments intact, which are crucial drivers of AD pathogenesis [65].

In short, these data support a model in which IL-5 functions as a tissue-level immune regulator rather than a unidimensional eosinophil growth factor. While both anti-IL-5 and anti-IL-5Rα therapies can recalibrate immune balance at the systemic level [55,56], emerging evidence suggests that broader attenuation of IL-5 signaling may influence multiple immune cell populations that contribute to chronic airway inflammation [57,58,61]. These observations raise the possibility that different modes of IL-5 pathway interruption engage distinct components of tissue inflammatory networks. Whether such differences translate into disease-specific clinical outcomes requires further direct investigation.

### 4.2. Does IL-5 Drive Airway Remodeling Solely by Sustaining Eosinophils, or Does It Directly Program Structural Cells Toward a Remodeling Phenotype?

IL-5 sits upstream of several structural changes that define chronic asthma, and the remodeling signal likely reflects more than the downstream consequences of eosinophil accumulation alone [66,67,68,69]. In a long-term OVA challenge model, IL-5-deficient mice developed markedly less peribronchial fibrosis and airway smooth muscle thickening, alongside lower indices of Transforming Growth Factor-β (TGF-β) activation in the airway wall, supporting a causal role for IL-5-dependent pathways in remodeling biology [66].

Consistent with that concept in humans, early interventional biopsy data showed that neutralizing IL-5 reduced deposition of extracellular matrix proteins in the bronchial subepithelial compartment in mild atopic asthma [68]. Whether this improvement is entirely secondary to eosinophil reduction has remained difficult to prove [67], but the “direct structural-cell” hypothesis has strengthened because human lung fibroblasts express IL-5Rα and respond to IL-5 with proliferative and pro-survival signaling (including ERK/Akt activation), which is the kind of intracellular wiring we would expect to feed into fibrosis and airway wall thickening [70].

More recently, mechanistic work has pushed this further by showing that IL-5 can directly program asthmatic-derived fibroblasts toward a remodeling phenotype—increasing collagen I and fibronectin expression, shifting Matrix Metalloproteinase (MMP)/Tissue Inhibitor of Metalloproteinases (TIMP) balance, inducing pro-fibrotic cytokines (including TGF-β), and reducing apoptosis, with transcriptomic signatures pointing to MAPK activation and apoptosis resistance [69]. And importantly, this “structural impact” is no longer just a short-term signal: The MESILICO (Mepolizumab in Severe eosinophiLIc asthma with fixed airflow obstruction: a COhort study) study provides rare longitudinal biopsy evidence that airway structural changes in severe asthma are not fixed, but can improve with sustained IL-5 blockade [71,72]. In patients with late-onset severe eosinophilic asthma and fixed airflow obstruction, mepolizumab treatment was associated with progressive and durable reductions in key histopathologic features of airway remodeling, including sub-basement membrane thickness, airway smooth muscle area and layer thickness, and epithelial damage [71]. These changes were already evident at 12 months and were maintained or further improved after 3 years of continuous therapy, alongside reductions in tissue eosinophil numbers [71]. Importantly, the magnitude of airway smooth muscle layer thinning correlated with improvements in lung function, linking structural modification to physiological benefit [72]. Together, these findings challenge the long-held assumption that airway remodeling is irreversible and support the concept that prolonged IL-5 neutralization can exert disease-modifying effects at the tissue level, beyond symptom control or suppression of circulating eosinophils [71,72].

### 4.3. How Do IL-5-Dependent Structural and Inflammatory Programs Intersect with Mucus Biology to Produce Persistent Airway Obstruction in Severe Asthma?

Although goblet cells are the principal source of airway mucins, mucus plugging in severe asthma reflects more than increased mucin production alone. Pathologic and imaging studies show that airway plugs are complex structures composed not only of gel-forming mucins but also of cellular debris, extracellular DNA, and inflammatory proteins that markedly increase mucus viscosity and resistance to clearance [46]. In this context, eosinophils emerge as critical contributors to mucus pathology. Elegant genetic evidence from eosinophil-deficient PHIL transgenic mice demonstrated that the absence of eosinophils protects against allergen-induced pulmonary mucus accumulation and airway hyperresponsiveness, despite intact goblet cell programs, establishing eosinophils as necessary drivers of mucus obstruction rather than passive bystanders [73].

IL-5 contributes to this process indirectly by sustaining eosinophil survival and activation within the airway lumen, thereby promoting epithelial injury and the release of eosinophil-derived granule proteins, extracellular traps, and Charcot–Leyden crystals that physically integrate into mucus plugs and increase their tenacity [46,74]. These eosinophil-associated components impair mucociliary clearance and favor airway obstruction even in the absence of proportional changes in goblet cell number or mucin gene expression [75]. Thus, while IL-13-dependent epithelial programs govern mucin production, IL-5 shapes mucus plugging by controlling the eosinophil-rich inflammatory environment in which mucus transitions from a protective secretion to a pathological airway plug [76]. This process establishes a vicious cycle: eosinophil-derived extracellular traps and Charcot–Leyden crystals incorporated into airway mucus perpetuate immune activation, intensify type 2 inflammation, and promote further eosinophil activation, leading to thicker, more tenacious mucus plugs and chronic airway obstruction [46,74,76,77].

Direct biophysical evidence links eosinophil-rich inflammation to pathologic mucus properties [78]. Surgically obtained nasal mucus from eosinophilic disease exhibits higher CT density, viscosity, dry weight, and hydrophobicity than non-eosinophilic mucus, with eosinophil-derived proteins strongly correlating with these physical characteristics. In vitro, aggregates formed by activated eosinophils through extracellular trap formation recapitulate these properties, and enzymatic disruption of extracellular traps reduces them, demonstrating that eosinophil-derived structures directly determine mucus quality rather than simply mucus quantity [78]. Clinically, this distinction helps explain why imaging-based mucus plug assessment has emerged as a meaningful disease marker [79,80,81]. Mepolizumab reduces airway mucus plug burden on CT in parallel with improvements in asthma control and lung function, with greater benefit observed in patients with higher baseline plug scores, supporting mucus plugging as a treatable eosinophil-linked trait in severe asthma [80,81].

## 5. IL-5 in COPD: Tissue-Restricted Eosinophilia and Context-Dependent Signaling

Viewed through the rEOS/iEOS framework, eosinophilic inflammation in COPD appears predominantly tissue-restricted and phenotypically distinct from asthma. Peripheral blood eosinophil counts correlate poorly with eosinophil numbers in bronchial tissue or BAL, limiting their value as a surrogate of lung-resident eosinophils [82]. Instead, activation markers such as Charcot–Leyden crystals/Galectin-10 are enriched within the airway lumen, consistent with locally retained, activated eosinophils. Tissue IL-5Rα expression tracks with lung eosinophil burden and exacerbation frequency but not with blood eosinophil counts, suggesting that IL-5 signaling in COPD may be more closely linked to tissue-level activity than systemic eosinophilia [82].

Transcriptomic analyses further distinguish eosinophilic COPD from asthma. Although subsets of COPD patients exhibit type 2-associated signatures, overlap with asthma-derived eosinophilic programs is limited, and only a small fraction of eosinophil-associated genes are shared between the two diseases [83,84]. In COPD, eosinophil presence associates preferentially with local epithelial and stromal programs, whereas canonical asthma markers such as Periostin and SERPINB2 are inconsistently expressed and strongly influenced by smoking status [85]. These findings support a COPD airway environment shaped by tissue adaptation rather than classic IL-5-driven iEOS expansion.

Mechanistic studies reinforce this distinction. Eosinophils accumulating in emphysematous lungs display transcriptomic enrichment of proteolytic pathways, particularly Cathepsin L, which contributes directly to extracellular matrix degradation and alveolar destruction [29,86,87]. While IL-5 neutralization reduces eosinophil survival in experimental models, inhibition of Cathepsin L alone attenuates tissue damage, indicating that IL-5 may function upstream of remodeling programs partially uncoupled from classic type 2 inflammation [86]. Clinically, eosinophil-derived Cathepsin L correlates with emphysema severity, supporting a role for eosinophils as long-lived tissue effectors rather than mucus-dominant inflammatory drivers in COPD [86].

Comparative biomarker studies further highlight divergent inflammatory logics between COPD and asthma. Soluble mediators, including arachidonic acid metabolites and IL-5, show distinct patterns across asthma, COPD, and ACO, with IL-5 in COPD not uniformly tracking with airflow limitation or disease severity [88]. Multi-omics profiling demonstrates that eosinophils in eosinophilic COPD and ACO exhibit signatures enriched for antiviral, metabolic, and innate immune pathways, rather than classical IL-5-dominant programs, and require combinatorial cytokine signaling for phenotypic reproduction in vitro [89]. Together, these data argue against direct extrapolation of asthma-derived IL-5 biomarker logic into COPD.

Collectively, eosinophilic inflammation in COPD reflects a context-dependent balance shaped by local tissue cues and mixed inflammatory environments. In this setting, IL-5 appears to act less as a systemic eosinophil amplifier and more as a permissive signal within a heterogeneous network, helping explain both the limitations of blood eosinophils as biomarkers and the variable clinical responses to IL-5-targeted therapies in COPD.

### Do Emerging Biomarker Profiles Reveal Fundamentally Different Inflammatory Logics in COPD and Asthma That Could Inform Precision Use of Biologic Therapies?

Building on the concept that eosinophilic inflammation in COPD is compartmentalized and biologically distinct from asthma, comparative biomarker studies across asthma, COPD, and asthma–COPD overlap (ACO) further underscore that superficially similar inflammatory labels often reflect different underlying mechanisms. Although airway inflammation is a shared feature across these conditions, the composition, regulation, and clinical meaning of inflammatory mediators differ substantially, complicating diagnosis and therapeutic extrapolation.

Early efforts to distinguish these disease states using soluble inflammatory markers focused on arachidonic acid metabolites, including cysteinyl leukotrienes (LTC_4_, LTD_4_, LTE_4_) and prostanoids such as prostaglandin D_2_ (PGD_2_) and prostaglandin E_2_ (PGE_2_). In a comparative clinical study of asthma, COPD, and ACO, PGD_2_ levels were significantly higher in asthma and ACO than in COPD, while PGD_2_, cysteinyl leukotrienes, and A disintegrin and metalloproteinase 33 (ADAM33) levels correlated negatively with airflow obstruction specifically in ACO. In contrast, IL-5 showed a distinct pattern in COPD, where higher circulating IL-5 levels correlated positively with multiple lung function parameters, suggesting that IL-5 biology in COPD may not uniformly track with airway obstruction or disease severity in the same way it does in asthma [88]. These findings already hint that IL-5 in COPD may reflect a compensatory or context-dependent signal, rather than a direct surrogate of pathogenic eosinophilic activity.

More granular insight comes from multi-omics profiling of eosinophils isolated from patients with eosinophilic COPD and ACO [89]. Transcriptomic and proteomic analyses reveal that eosinophils in these settings exhibit signatures enriched for antiviral responses, cholesterol metabolism, and innate immune signaling, rather than the classical IL-5-dominant survival and activation programs seen in asthma. Importantly, these eosinophil phenotypes could only be reproduced in vitro when IL-5 signaling occurred in combination with IL-33, TNF-α, and IFN-γ, indicating that IL-5 acts as a contextual amplifier rather than a solitary driver in COPD-related eosinophilia [89]. Consistent with this, lipidomic analyses showed impaired synthesis of cyclooxygenase-derived mediators such as PGE_2_ in eosinophilic COPD, further differentiating it from asthma-associated Type 2 inflammation [89].

Phenotypic studies comparing blood and sputum eosinophils reinforce this divergence. While blood eosinophils from asthma and COPD patients display broadly similar surface marker expression, sputum eosinophils in COPD exhibit a distinct activation profile, characterized by increased CD193^+^ and CD66b^+^ subsets, in contrast to asthma, where CD11b^+^ eosinophils predominate in the airway lumen [90]. These observations align with the rEOS/iEOS framework, suggesting that eosinophils in COPD are more likely shaped by local tissue cues and mixed inflammatory environments, rather than being uniformly IL-5-dependent circulating effector cells.

Serum biomarker studies further illustrate that even when eosinophilia is present in both diseases, the upstream regulatory balance differs. Compared with severe eosinophilic asthma, patients with eosinophilic COPD exhibit higher levels of soluble IL-5Rα, MET, and markers of matrix turnover, alongside reduced eotaxin-1 and FcεRI signaling. While IL-5 and IL-25 remain part of the inflammatory milieu, their association with disease appears embedded within a broader imbalance between Type 1 and Type 2 pathways, rather than a dominant IL-5-centric axis [91].

These data argue against a simplistic transfer of asthma-derived eosinophil biomarkers or IL-5-based treatment logic into COPD. Instead, they support a model in which IL-5 participates in COPD as a modulatory signal within a heterogeneous inflammatory network, influencing eosinophil phenotype, survival, and function in concert with viral, metabolic, and innate immune pathways.

## 6. Clinical Trial Landscape of IL-5-Targeted Therapies Across Airway Diseases

Therapies directed against the IL-5 pathway interrupt this axis in two distinct ways. Mepolizumab and Reslizumab bind circulating IL-5, preventing interaction with the IL-5 receptor complex and thereby reducing downstream survival and activation signals in responsive cells [92,93]. In contrast, Benralizumab targets IL-5Rα on the cell surface and induces antibody-dependent cellular cytotoxicity, resulting in rapid and near-complete depletion of eosinophils and basophils [62]. Although these agents were developed to target the IL-5–eosinophil axis, their modes of pathway interruption are biologically distinct. Indirect treatment comparisons in severe eosinophilic asthma indicate that all three agents reduce exacerbations versus placebo across similar blood eosinophil strata, with some analyses suggesting greater improvements in exacerbation reduction and asthma control with mepolizumab; however, these findings should be interpreted cautiously in the absence of head-to-head trials [94].

To place these differences in context, we summarize pivotal trials of IL-5-targeted biologics across severe eosinophilic asthma, COPD, and CRSwNP, with EGPA and HES included as a related systemic eosinophilic condition. In severe asthma, IL-5 pathway inhibition consistently reduces exacerbations and oral corticosteroid exposure [95]. In EGPA and HES, IL-5-targeted therapies, including Mepolizumab and Benralizumab, have demonstrated improvements in remission rates, reduction in disease activity, and steroid-sparing effects compared with placebo in selected patient populations [96,97,98,99]. In COPD and CRSwNP, however, results have been more variable, with benefit observed in selected populations but less consistent effects on primary endpoints overall [38,100,101,102,103,104,105,106]. The relative contribution—or “weight”—of eosinophils to disease pathogenesis appears to differ across these conditions.

The intent of this comparison (Table 2) is descriptive rather than comparative. Differences in study design, inclusion criteria, and endpoints preclude direct cross-trial conclusions. Nonetheless, when viewed alongside emerging data on tissue-level signaling, receptor biology, and inflammatory context, the trial landscape underscores that interventions aimed at the same biological axis do not necessarily produce uniform clinical effects across airway diseases.

Table 2 summarizes Phase III randomized controlled trials of IL-5 ligand-neutralizing antibodies (mepolizumab, reslizumab) and the IL-5 receptor α-targeting antibody benralizumab across severe asthma, CRSwNP, EGPA, HES, and COPD. Asthma trials included are limited to those with AER as the primary endpoint. The table presents mechanism of action, dosing strategy, key inclusion criteria, and primary outcomes; “Primary met” denotes achievement of the prespecified primary endpoint, and INF design is specified where applicable.

## 7. Mechanistic Considerations and Emerging Areas for Investigation in IL-5-Targeted Therapy (Figure 1)

This schematic first highlights reports of IL-5Rα expression on selected immune and structural cell populations beyond eosinophils, with their detailed mechanisms in human airway disease warranting further exploration. It then depicts classical IL-5-mediated signaling in eosinophils, where IL-5 binding to the membrane IL-5 receptor (mIL-5R; IL-5Rα/βc) supports survival and activation. Distinct eosinophil subsets (rEOS and iEOS) with differential IL-5 responsiveness are illustrated. Soluble IL-5R (sIL-5R) is shown as a potential modulator of IL-5 bioavailability. Downstream tissue features associated with IL-5-linked inflammation are summarized.

**Figure 1:**
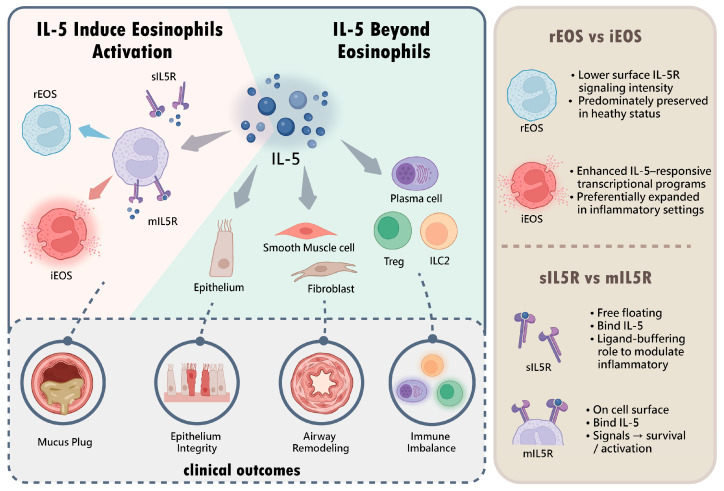
Mechanistic considerations and emerging areas for investigation in IL-5-targeted therapy.

This section therefore organizes key mechanistic perspectives into two complementary domains: pathways primarily centered on granulocyte biology and those reflecting broader tissue and immune network regulation beyond a single-cell focus.

### 7.1. Granulocyte-Centered Considerations

- Heterogeneity of activation states across diseases: Eosinophil activation states vary across airway diseases and coincide with shifts in the relative balance between type 2 and non-type 2 inflammatory gene networks, although direct mechanistic links remain to be established.

In asthma, eosinophils in both blood and tissue frequently display strong dependence on IL-5 signaling, and reductions in eosinophil numbers following IL-5 pathway inhibition are often associated with clinical benefit [31]. In contrast, studies in COPD and CRSwNP consistently show weaker correlations between blood eosinophil counts and eosinophil burden within airway tissues or bronchoalveolar compartments. In these settings, tissue IL-5Rα expression and local eosinophil activation markers appear to track more closely with disease activity and exacerbation risk than circulating eosinophil counts [82,83,114]. Transcriptomic analyses further indicate that eosinophils in COPD and CRSwNP exhibit disease-specific activation states shaped by local epithelial, stromal, and environmental cues, rather than uniform IL-5-dominant inflammatory programs [83,85].

Receptor isoform balance: Soluble versus membrane-bound IL-5 receptor isoforms may modulate pathway engagement.

Regulation of IL-5 signaling adds another layer of complexity. Beyond cellular distribution of IL-5Rα, regulation of IL-5 signaling is further shaped by the balance between membrane-anchored and soluble IL-5 receptor isoforms. Alternative splicing of IL5RA generates a soluble IL-5Rα variant that binds IL-5 with high affinity but lacks signaling capacity, thereby functioning as a decoy receptor that limits ligand availability and tunes downstream signaling within tissue compartments rather than inducing cell depletion [115,116,117]. Consistent with this regulatory model, bronchial biopsy studies in asthma demonstrate coordinated upregulation of both membrane-bound and soluble IL-5Rα on airway eosinophils, with membrane IL-5Rα expression inversely correlating with FEV_1_, while soluble IL-5Rα expression shows a positive association with lung function, indicating that receptor isoform balance—not eosinophil number alone—shapes the physiological impact of IL-5 signaling in vivo [118]. Elevated soluble IL-5Rα has been documented in multiple eosinophilic airway diseases, including nasal polyposis and acute exacerbations of COPD, where levels increase locally in tissue, nasal secretions, sputum, and serum and track with disease activity and lung function recovery rather than smoking exposure or absolute eosinophil counts [119,120,121]. Experimental and clinical studies further demonstrate that soluble IL-5Rα can attenuate IL-5-driven eosinophil activation without inducing cytotoxic depletion, consistent with a ligand-buffering role that modulates inflammatory tone rather than cell number [122,123,124]. In this context, IL-5 ligand neutralization (e.g., mepolizumab) is positioned to suppress signaling across both membrane-bound and soluble receptor pools, whereas IL-5Rα-directed ADCC strategies depend strictly on surface receptor density and susceptibility to immune-mediated killing, rendering them insensitive to soluble receptor-mediated buffering. This distinction offers a biologically grounded explanation for why IL-5 ligand blockade can modulate tissue inflammation even when eosinophil depletion is incomplete, while receptor-directed cytotoxic approaches may fail to fully disrupt IL-5-dependent signaling networks in diseases dominated by tissue-resident immune programs such as CRSwNP and selected COPD endotypes.

Implications for ligand neutralization vs. receptor-directed cytotoxicity: Receptor-directed cytotoxic strategies may be influenced by host immune context.

IL-5Rα-directed antibody-dependent cellular cytotoxicity relies on sufficient surface receptor expression and intact effector-cell function, particularly NK-cell activity [125]. In chronic inflammatory states or in patients receiving immunosuppressive therapies, effector-cell number or function may be variably altered. Observations from selected clinical contexts, such as EGPA populations receiving concomitant immunosuppression, raise the possibility that host immune context could influence the effectiveness of ADCC-based strategies, although direct evidence remains limited and largely indirect [96].

### 7.2. Broader Tissue and Immune Network Context

- IL-5 signaling may extend beyond eosinophils to engage additional immune and structural cell populations in inflamed airway tissues

As outlined above, IL-5 biology in airway disease is not limited to eosinophil differentiation and survival. Functional IL-5Rα expression has been described on multiple immune and structural cell populations within diseased airway tissue—including epithelial cells, plasma cells, mast cells, basophils, selected innate immune subsets, and fibroblasts [9,42,57,58,61,70]. Evidence from CRSwNP provides a translational context for this concept. Benralizumab achieves rapid and sustained depletion of circulating and tissue eosinophils, with reductions in eosinophil-derived mediators. In contrast, most other immune cell populations (e.g., NK cells, neutrophils, CD4^+^/CD8^+^ T cells, basophils, mast cells) and key cytokines, soluble IL-5Rα, or IgE appear largely unchanged, suggesting limited impact on broader tissue inflammatory architecture [37].

Similarly, near-complete eosinophil depletion induced by a non-IL-5-directed agent (dexpramipexole) is not accompanied by meaningful reductions in polyp burden or consistent clinical improvement [36]. In parallel, the phase III ORCHID study evaluating Benralizumab in CRSwNP was discontinued prior to completion, and detailed peer-reviewed efficacy data have not been reported [38]. Based on publicly available summary data, the mean (SD) change in nasal polyp score (NPS) at Week 56 was −0.3 (1.6) in the benralizumab arm versus −0.1 (1.3) with placebo. Given the limited reporting and early discontinuation, definitive conclusions regarding clinical efficacy cannot be drawn [38].

Taken together, these considerations may offer one framework for interpreting the differing clinical patterns observed with IL-5 ligand neutralization and IL-5Rα-directed cytotoxic approaches across airway diseases. These observations remain hypothesis-generating, as existing trials were not designed to directly evaluate tissue-level IL-5 signaling, receptor isoform dynamics, non-eosinophil target engagement, or effector-cell competence.

## 8. Reframing Treatment Goals: From Pathway Inhibition to Disease Control

Across immune-mediated inflammatory diseases, treatment goals have shifted from blocking individual pathways toward broader concepts of disease control, integrating symptoms, exacerbations, functional status, and tissue-level outcomes [126,127,128]. Increasing emphasis is placed less on cytokine suppression itself and more on sustained clinical stability. In airway diseases, this evolution is reshaping how success is defined. In severe asthma, clinical remission—freedom from exacerbations, minimal oral corticosteroid use, stable lung function, and sustained symptom control—is increasingly discussed [129,130]. In CRSwNP, outcomes are framed around durable polyp control and prevention of recurrence, reflecting its chronic inflammatory nature [131]. In COPD, goals remain centered on stability—reducing exacerbations and preserving lung function—although biomarker-informed strategies indicate movement toward more individualized care [132].

Within this landscape, IL-5-targeted therapies are best interpreted not solely as eosinophil-depleting agents, but as interventions whose impact must be evaluated against broader disease-control metrics. The treatable-traits framework conceptualizes airway diseases as interacting biological and clinical processes rather than dominance of a single inflammatory axis [133,134]. In this context, the clinical effects of IL-5 pathway interruption may depend on how effectively tissue-level inflammatory and structural programs are modulated across asthma, CRSwNP, and COPD [51,52,117]. This perspective accommodates heterogeneity in response and recognizes that different modes of targeting IL-5 may engage distinct components of airway immune networks, without presuming uniform benefit across diseases or patient populations.

## 9. Conclusions

In summary, while the biological axis between IL-5 and eosinophils remains fundamentally stronger and more clinically validated than its connection with any other cell type, this review reframes IL-5 as a pleiotropic coordinator rather than a unidimensional growth factor. By integrating evidence of functional IL-5Rα expression on epithelial cells, fibroblasts, and diverse immune subsets, we illustrate a transition toward network-informed disease control. Recognizing that IL-5 shapes the airway microenvironment through structural remodeling and mucus pathology—acting either in concert with or through pathways independent of systemic eosinophil counts—provides a mechanistic basis for the heterogeneous clinical responses observed in practice. Ultimately, achieving durable disease control in severe airway diseases requires a broader conceptual shift: viewing IL-5 as a context-dependent regulator within a dynamic tissue network. Future research must further delineate the in vivo independence of these non-canonical signals to fully realize the potential of precision medicine in eosinophilic pathologies [135,136,137,138,139,140,141].

## Figures and Tables

**Table 1:** Phenotypic and functional distinction between resident and inflammatory eosinophil subsets in human and murine studies.

Species/Study Type Population/Disease Context	Feature	Resident Eosinophils (rEOS)	Inflammatory Eosinophils (iEOS)
Human-SEA stratum			
Healthy donors [32]	Primary Surface Marker	CD62L^bright^ (L-selectin high)	CD62L^low^ (L-selectin low)
	Proportion in blood	Predominant population	Very low frequency
	IL-5 receptor (CD125)	Lower expression	Relative IL-5Rα expression higher on CD62L low subset
	Response to IL-5 (in vitro)	IL-5 promotes CD62L downregulation, increasing the proportion of CD62L^low^ cells	Expanded by IL-5 stimulation
	Effect of anti-IL-5 (in vitro)	Blocks IL-5-induced expansion of CD62L^low^ eosinophils and prevent IL-5-mediated downregulation of CD62L	IL-5-induced expansion prevented by Mepolizumab
	Functional profile	Homeostatic/regulatory phenotype	Minimal inflammatory role at steady state
	Clinical correlation	Not disease-associated	Not expanded in health
SEA (bio-naïve) [32]	Relative proportion	Reduced relative proportion	Relative enrichment of CD62L^low^ eosinophils
	Asthma control (ACT/ACQ-5)	Higher ACT, lower ACQ-5	Inversely associated with ACT score; positively with ACQ-5
	Annual exacerbations	Lower frequency	Associated with exacerbation frequency
SEA on anti-IL5 (Mepolizumab) short-term [32]	Subset redistribution	Increased proportion	Reduction in CD62L^low^ proportion
	Clinical response	Improvement parallels rEos’ predominance	Reduction correlates with ΔACT and ΔACQ-5
SEA on anti-IL5 (Mepolizumab) long-term [32]	Durability	Stable predominance	Persistently low over time
Human cross-disease cohort			
Healthy vs. asthma vs. COPD [30]	Proportion in healthy subjects (HSs)	Predominant subtype	Very low proportion (~0.6%)
	Proportion in asthma	Dominant population (~70–75%)	Relative enrichment vs. Healthy subjects (~25–30%)
	Proportion in COPD	Predominant subtype	Very low proportion (~0.5–0.7%)
	Relation to COPD severity	No clear association (GOLD grade)	No clear association (GOLD grade)
	Therapeutic implication	Likely preserved homeostatic pool	Plausibly IL-5-responsive
Human-Ex vivo blood eosinophil subset functional study			
Asthma context [31]	Dominant subtype	Severe non-allergic eosinophilic asthma (SNEA)	Allergic asthma
	Adhesion to ASM cells	Higher adhesion	Lower adhesion
	Baseline survivability (24 h)	Lower than iEOS	Higher baseline survival compared with rEOS
	Survivability after ASM interaction	Marked survival increase	Minimal additional gain
Murine-Lung Model			
Lung Model [33]	Localization	Parenchymal (Deep Tissue Residence)	Peribronchial (airway infiltration)
	Nuclear morphology	Ring-shaped nucleus	Segmented nucleus
	Surface markers	Siglec-F^int^ CD62L^+^ CD101^low^	Siglec-F^hi^ CD62L^−^ CD101^hi^
	IL-5 dependency	IL-5 independent	IL-5 dependent
	Role in allergy	Regulatory; suppress Th2 responses	Pro-inflammatory
	Effect on DCs	Inhibits DC maturation	No regulatory effect

**Table 2:** Clinical trial landscape: IL-5 ligand vs. IL-5Rα targeting.

Disease	Drug (MoA)	Trial	Route (Dosing Strategy)	Key Inclusion Criteria	Primary Endpoint	Interpretation Tag
Severe asthma	Benralizumab (anti-IL-5Rα/ADCC)	CALIMA [107]	SC (fixed dose)	Med–high-dose ICS + LABA; ≥2 exacerbations in previous year; Stratified by baseline blood eosinophils (thresholds prespecified by trial).	AER vs. PLB (population: high-dose ICS + LABA and BEC ≥ 300 cells/μL)	Primary Met
Severe asthma	Benralizumab (anti-IL-5Rα/ADCC)	SIROCCO [108]	SC (fixed dose)	High-dose ICS + LABA; ≥2 exacerbations in previous year; Stratified by baseline blood eosinophils (thresholds prespecified by trial).	AER vs. PLB (Population: High-dose ICS + LABA and BEC ≥ 300 cells/μL)	Primary Met
Severe asthma	Mepolizumab (anti-IL-5)	DREAM [109]	IV (fixed dose)	BEC ≥ 300 cells/μL or sputum ≥ 3%; ICS ≥ 880 μg fluticasone propionate equivalent per day + controllers; ≥2 exacerbations in previous year.	AER vs. PLB	Primary Met
Severe asthma	Mepolizumab (anti-IL-5)	MENSA [110]	SC (fixed dose)	BEC ≥ 150 at screening or ≥300 cells/μL in previous year; High-dose ICS + another controller; ≥2 exacerbations in previous year.	AER vs. PLB	Primary Met
Severe asthma	Reslizumab (anti-IL-5)	NCT01287039 (study 1) and NCT01285323 (study 2) [111]	IV (weight-based dosing)	BEC ≥ 400 cells/μL at screening; Med–high-dose ICS; ≥1 exacerbation in previous year.	AER vs. PLB	Primary Met
Severe asthma	Reslizumab (anti-IL-5)	NCT02452190 (study 1) [112]	SC (fixed dose)	BEC ≥ 300 cells/μL at screening; Med–high-dose ICS + another controller; ≥2 exacerbations in previous year.	AER vs. PLB	Primary not met
CRSwNP	Benralizumab (anti-IL-5Rα/ADCC)	OSTRO [100]	SC (fixed dose)	NPS ≥ 5 (Min. 2 per side); Mean NBS ≥ 1.5; SNOT-22 ≥ 30; Stable INCS; Prior SCS or surgery.	Co-primary endpoints: ΔNPS and ΔNBS vs. PLB	Primary met
CRSwNP	Benralizumab (anti-IL-5Rα/ADCC)	ORCHID [38]	SC (fixed dose)	NPS ≥ 5 (Min. 2 per side); Mean NBS ≥ 1.5; SNOT-22 ≥ 20; Stable INCS; Prior SCS or surgery; BEC of >2% or ≥150/μL at enrolment.	Co-primary endpoints: ΔNPS and ΔNBS vs. PLB	Inconclusive (study discontinued; limited publicly available data)
CRSwNP	Mepolizumab (anti-IL-5)	SYNAPSE [101]	SC (fixed dose)	NPS ≥ 5 (Min. 2 per side); VAS obstruction > 5; Overall VAS > 7; ≥1 surgery in the last 10 years; Stable INCS.	Co-primary endpoints: ΔNPS and mean nasal obstruction VAS vs. PLB	Primary met
EGPA	Benralizumab (anti-IL-5Rα/ADCC)	MANDARA (vs. Mepolizumab) [96]	SC (fixed dose)	BEC > 1000 cells/μL and/or >10% of eosinophils; Stable OCSs (7.5–50 mg/day); Relapsing or refractory EGPA.	Remission vs. Mepolizumab	Primary met (INF design)
EGPA	Mepolizumab (anti-IL-5)	MIRRA [97]	SC (fixed dose)	BEC > 1000 cells/μL or >10% of eosinophils; Stable OCS (7.5–50 mg/day); Relapsing or refractory EGPA.	Remission vs. PLB	Primary met
HES	Benralizumab (anti-IL-5Rα/ADCC)	NATRON [113]	SC (fixed dose)	*FIP1L1-PDGFRA*-negative HES; With flare signs/symptoms at screening or ≥2 flares within the past year; Corticosteroid-responsive.	Time to first HES flare	Primary met
HES	Mepolizumab (anti-IL-5)	Roufosse et al. [98]	SC (fixed dose)	*FIP1L1-PDGFRA*-negative HES; >2 flares within the past 12 months and BEC ≥ 1000 cells/μL at screening; Receiving stable background therapy.	% of patients who experienced a flare	Primary met
COPD	Benralizumab (anti-IL-5Rα/ADCC)	GALATHEA [102]	SC (fixed dose)	Dual or triple inhaled therapy; ≥2 moderate or ≥1 severe exacerbation in previous year; Post-BD FEV_1_ 20–65% predicted; Stratified by baseline blood eosinophils (thresholds prespecified by trial).	AER vs. PLB (Population: Blood EOS ≥ 220 cells/μL)	Primary not met
COPD	Benralizumab (anti-IL-5Rα/ADCC)	TERRANOVA [102]	SC (fixed dose)	Dual or triple inhaled therapy; ≥2 moderate or ≥1 severe exacerbation in previous year; Post-BD FEV_1_ 20–65% predicted; Stratified by baseline blood eosinophils (thresholds prespecified by trial).	AER vs. PLB (Population: Blood EOS ≥ 220 cells/μL)	Primary not met
COPD	Benralizumab (anti-IL-5Rα/ADCC)	RESOLUTE [105]	SC (fixed dose)	BEC ≥ 300 cells/μL; Triple inhaled therapy; ≥2 moderate or ≥1 severe exacerbation in previous year; Post-BD FEV_1_ 20–65% predicted.	AER vs. PLB	Primary not met
COPD	Mepolizumab (anti-IL-5)	METREX [103]	SC (fixed dose)	High stratum (BEC ≥ 150 at screening OR ≥ 300 cells/μL in past year) OR Low stratum (BEC < 150 cells/μL at screening); Triple inhaled therapy; ≥2 moderate or ≥1 severe exacerbation in previous year; Post-BD FEV_1_ 20–80% predicted.	AER vs. PLB	Primary not met overall; prespecified eosinophilic subgroup showed benefit (High stratum)
COPD	Mepolizumab (anti-IL-5)	METREO [103]	SC (fixed dose) [100 mg or 300 mg]	BEC ≥ 150 at screening OR ≥ 300 cells/μL in past year; Triple inhaled therapy; ≥2 moderate or ≥1 severe exacerbation in previous year; Post-BD FEV_1_ 20–80% predicted.	AER vs. PLB	Primary not met
COPD	Mepolizumab (anti-IL-5)	MATINEE [104]	SC (fixed dose)	BEC ≥ 300 (screening) AND ≥ 150 cells/μL (past year); Triple inhaled therapy; ≥2 moderate or ≥1 severe exacerbation in previous year; Post-BD FEV_1_ 20–80% predicted.	AER vs. PLB	Primary Met

## Data Availability

No new data were created or analyzed in this study. Data sharing is not applicable to this article.

## Abbreviations

IL-5, interleukin-5; IL-5R, interleukin-5 receptor; mIL-5R, membrane IL-5 receptor; sIL-5R, soluble IL-5 receptor; rEOS, resident eosinophils; iEOS, inflammatory eosinophils; ILC2, group 2 innate lymphoid cells; Treg, regulatory T cells; SEA: severe eosinophilic asthma; COPD, chronic obstructive pulmonary disease; IL-5Rα/CD125, interleukin-5 receptor alpha; ACT, Asthma Control Test; ACQ-5, Asthma Control Questionnaire-5; ICS, inhaled corticosteroid; ASM, airway smooth muscle; DC, dendritic cell; Th2, T helper type 2; Siglec-F, sialic acid-binding immunoglobulin-like lectin F; CD62L, L-selectin; ADCC, antibody-dependent cellular cytotoxicity; AER, annualized exacerbation rate; BEC, blood eosinophil count; post-BD, post-bronchodilator; COPD, chronic obstructive pulmonary disease; CRSwNP, chronic rhinosinusitis with nasal polyps; EGPA, eosinophilic granulomatosis with polyangiitis; EOS, eosinophils; HES, hypereosinophilic syndrome; ICS, inhaled corticosteroids; IL-5Rα, interleukin-5 receptor alpha; INF, non-inferiority; INS, intranasal corticosteroids; IV, intravenous; LABA, long-acting β2-agonist; LAMA, long-acting muscarinic antagonist; Min., minimal; MoA, mechanism of action; NBS, Nasal Blockage Score; NP, nasal polyp; NPS, nasal polyp score; OCS, oral corticosteroids; PLB, placebo; SC, subcutaneous; SCS, systemic corticosteroids; SoC, standard of care; VAS, visual analogue scale.
